# Value of TCT combined with serum CA153 and CA50 in early diagnosis of cervical cancer and precancerous lesions

**DOI:** 10.12669/pjms.38.6.5503

**Published:** 2022

**Authors:** Heyue Li, Linxia Li, Jianming Sun, Shengdong Dong, Hong Li

**Affiliations:** 1 Heyue Li, Division of Urological and Reproductive Medicine, Shanghai 7th People’s Hospital Affiliated to Shanghai University of Traditional Chinese Medicine, Shanghai 200137, China; 2 Linxia Li, Division of Urological and Reproductive Medicine, Shanghai 7th People’s Hospital Affiliated to Shanghai University of Traditional Chinese Medicine, Shanghai 200137, China; 3 Jianming Sun, Division of Urological and Reproductive Medicine, Shanghai 7th People’s Hospital Affiliated to Shanghai University of Traditional Chinese Medicine, Shanghai 200137, China; 4 Shengdong Dong, Division of Urological and Reproductive Medicine, Shanghai 7th People’s Hospital Affiliated to Shanghai University of Traditional Chinese Medicine, Shanghai 200137, China; 5 Hong Li, Division of Urological and Reproductive Medicine, Shanghai 7th People’s Hospital Affiliated to Shanghai University of Traditional Chinese Medicine, Shanghai 200137, China

**Keywords:** Cervical cancer, Thinprep cytologic test, Carbohydrate antigen 153, Carbohydrate antigen 50, Early diagnosis

## Abstract

**Objectives:**

To determine the application value of thinprep cytologic test (TCT) combined with serum carbohydrate antigen 153 (CA153) and carbohydrate antigen 50 (CA50) detection in the early diagnosis and screening of cervical cancer and precancerous lesions.

**Methods:**

A total of 187 females with cervical lesions admitted to Shanghai 7th People’s Hospital Affiliated to Shanghai University of Traditional Chinese Medicine from January 2017 to December 2018 were selected and divided into two groups: the cervical cancer group and the cervical precancerous lesion group, with 16 cases in the cervical cancer group and 171 cases in the cervical precancerous lesion group (cervical precancerous lesions were divided into 63 cases of the CNI group, 59 cases of the CNII group and 49 cases of the CNIII group). During the same period, 106 healthy females were selected as the healthy group. The serum tumor markers CA153 and CA50 of all subjects were detected by chemiluminescence method; The diagnostic value of TCT combined with serum CA153 and CA50 in cervical cancer and precancerous lesions was analyzed with colposcopy pathological diagnosis results as gold standard; ROC curve was drawn to evaluate the diagnostic value of serum TCT, CA153 and CA50 in cervical cancer and precancerous lesions.

**Results:**

The levels of serum CA153 and CA50 in the cervical cancer group were significantly higher than those in the cervical precancerous lesion group and the healthy group (p< 0.05), and the levels of serum CA153 and CA50 in the cervical precancerous lesion group were significantly higher than those in the healthy group (p< 0.05). The sensitivity of TCT, serum CA153 and serum CA50 in the single detection of cervical cancer and precancerous lesions was 95.93%, 97.54% and 96.00%, the specificity was 59.41%, 60.23%, 60.12%, the accuracy was 74.74%, 75.77%, 75.43%, the positive predictive value was 62.03%, 63.64%, 63.10%, and the negative predictive value was 96.22%, 97.17% and 95.28%, respectively. The sensitivity, specificity, accuracy, positive predictive value and negative predictive value of TCT combined with serum CA153 and CA50 were 96.77%, 73.19%, 85.67%, 80.21% and 95.28%, respectively. ROC curve showed that the area under the curve (AUC) of TCT and serum CA153 and CA50 in the single detection of cervical cancer and precancerous lesions was 0.791, 0.864 and 0.787, respectively, the AUC of combined detection of TCT and serum CA153 and CA50 in patients with cervical cancer and precancerous lesions was 0.877, which was significantly higher than that of single detection (p< 0.05).

**Conclusions:**

TCT combined with serum CA153 and CA50 has been reported as a treatment regimen with high accuracy, which has a high diagnostic efficiency for early diagnosis of cervical cancer and precancerous lesions, and can significantly improve the sensitivity.

## INTRODUCTION

On average, more than half a million women worldwide develop cervical cancer each year and nearly 300,000 die from it. A rising trend can be seen in the incidence and mortality of cervical cancer in recent years, and more and more young patients are being wantonly affected, which seriously threatens the life safety of females. Cervical cancer has become a public health problem that the country attaches great importance to and pays close attention to.^1^-^3^ Thinprep cytologic test (TCT) boasts simplicity of operation and is often used as an important means of clinical detection and screening for cervical cancer and precancerous lesions, which is capable of significantly improving the accuracy of the detection. However, the specificity and sensitivity of TCT are still relatively low.^4^-^6^ Serum carbohydrate antigen 153 (CA153) and carbohydrate antigen 50 (CA50), as tumor markers, can be used for joint detection to improve the accuracy of disease diagnosis. It has been applied in the early diagnosis of diseases such as cervical cancer, endometrial cancer, breast cancer and rheumatoid arthritis.^7^-^8^ In this study, the value of TCT combined with serum CA153 and CA50 detection in the early diagnosis of cervical cancer and precancerous lesions was analyzed.

## METHODS

A total of 187 females who received diagnosis and treatment for cervical lesions in Shanghai 7th People’s Hospital Affiliated to Shanghai University of Traditional Chinese Medicine from January 2017 to December 2018 were selected. Among them, 16 patients with cervical cancer were enrolled in the cervical cancer group, aged 27-66 years old, with an average age of (47.17 ± 5.77) years old. 171 patients with cervical lesions were enrolled in the Cervical precancerous lesion group, aged 25-63 years, with an average age of (44.55 ± 6.13) years. During the same period, 106 women who had undergone health examinations were selected as the health group, aged 22-69 years old, with an average age of (46.35 ± 5.79) years old. All the 171 patients with cervical precancerous lesions were further divided into the CNI group (63 cases), the CNII group (59 cases), and the CNIII group (49 cases).

### Ethical Approval

The study was approved by the Institutional Ethics Committee of Shanghai 7th People’s Hospital Affiliated to Shanghai University of Traditional Chinese Medicine on February 17,2017(No.:2017026), and written informed consent was obtained from all participants.

### Inclusion Criteria:

• Patients who had not undergone hysterectomy or cervical conization;
• Patients without a history of pelvic radiotherapy;
• Married female patients.

### Exclusion criteria:

• Patients with abnormal cytological tests;
• Patients with vaginal medication experience 72 h prior to detection;
• Patients complicated with other malignant tumors.^9^

All subjects were prohibited from sexual life, vaginal irrigation and vaginal medication within 60 h prior to the detection of TCT and serum tumor markers CA153 and CA50. Females who are menstruating should be tested 72 hour after the end of their period.

### TCT Detection

A speculum was used by the examinee to expose the cervix and the mucus on the surface of the cervix was wiped away. A special brush for liquid-based cell collection was utilized to rotate the cervix at a position of 1-2 cm for six times clockwise, and was immediately shaken and rinsed in the cell preservation solution, and sent for pathological examination. The smear was prepared using the ThinPrep automatic cell production machine and stained with the Prep2000 system before being interpreted. Cytological abnormalities include: (1) Definite atypical cervical squamous cells; (2) Atypical squamous cells with high-grade intraepithelial lesions were not excluded; (3) High-grade squamous cell intraepithelial lesions; (4) Low-grade squamous cell intraepithelial lesions; (5) Atypical glandular cells.^10^ TCT detection results of intraepithelial lesions or malignant cells were determined as positive, the rest were determined as negative.

### Serum Tumor Markers

CA153 and CA50 detection: The chemiluminescence method was used to detect the serum tumor marker CA153 and CA50 respectively, and the specific procedures were referred to the chemiluminescence kit of Beijing Putian Tongchuang Biotechnology Co., Ltd.

### Diagnostic Criteria

The diagnostic value of TCT combined with serum CA153 and CA50 in cervical cancer and precancerous lesions was analyzed with colposcopy biopsy results as the gold standard. Sensitivity = true positive cases/(true positive cases + false negative cases) × 100%; Specificity = true negative cases/(true negative cases + false positive cases) × 100%; Positive predictive value = true positive cases/(true positive cases and false positive cases) X 100%, negative predictive value = true negative cases/(true negative cases + false negative cases) × 100%; Accuracy = (true positive + true negative)/(true positive + false positive + true negative + false negative) × 100%.

### Statistical Analysis

All data in this study were statistically analyzed using SPSS 22.0, counting data were represented by “%”, and the chi-square test and Fisher’s exact probability method were performed; The measurement data were expressed as mean ± standard deviation (), and t test was performed. The ROC curve was drawn to evaluate the diagnostic value of blood TCT and serum CA153 and CA50 in cervical cancer and precancerous lesions. *p*<0.05 indicates a statistically significant difference.

## RESULTS

The levels of serum CA153 and CA50 in the cervical cancer group were significantly higher than those in the Cervical precancerous lesion group and the healthy group (*p*< 0.05), while the levels of serum CA153 and CA50 in the Cervical precancerous lesion group were significantly higher than those in the healthy group (*p*< 0.05). Table-I.

**Table I:** Comparison of the levels of serum CA153 and CA50 among the three groups.

Group	CA153 detection (ng/mL)	CA50 detection (ng/mL)
Cervical cancer group (n=16)	37.57±6.42^①②^	36.44±5.78^①②^
Cervical precancerous lesion group (n=171)	21.11±3.15^①^	19.87±3.47^①^
Healthy group (n=106)	8.34±1.21	7.99±1.34
F	1035.766	846.706
P	0.000	0.000

***Note***: ^①^ p< 0.05 compared with the healthy group;

^②^ p< 0.05 compared with the cervical precancerous lesion group.

The diagnosis result of pathological biopsy under colposcopy was used as the gold standard. When TCT, serum CA153 and serum CA50 were used separately, the sensitivity of the three groups was 95.93%, 97.54% and 96.00%, the specificity was 59.41%, 60.23%, 60.12%, the accuracy was 74.74%, 75.77%, 75.43%, the positive predictive value was 62.03%, 63.64%, 63.10%, and the negative predictive value was 96.22%, 97.17% and 95.28%, respectively. Table-II. The sensitivity, specificity, accuracy, positive predictive value and negative predictive value of TCT combined with serum CA153 and CA50 in the detection of cervical cancer and precancerous lesions were 96.77%, 73.19%, 85.67%, 80.21% and 95.28%, respectively. The specificity, accuracy and positive predictive value of cervical cancer and precancerous lesions were the highest when TCT was combined with serum CA153 and CA50, while the sensitivity and negative predictive value of cervical cancer and precancerous lesions were the highest when serum CA153 detection was conducted. Table-III and Table-IV.

**Table II:** Results of TCT, serum CA153 and CA50 alone or in combination on cervical cancer and precancerous lesions.

Group		TCT detection	CA153 detection	CA50 detection
Comparison of single detection between the three groups (positive/negative)	Cervical cancer group (n=16)	13/3	11/5	12/4
	Cervical precancerous lesion group (n=171)	103/68	108/63	106/65
	Healthy group (n=106)	4/102	3/103	5/101
	Total	120/173	122/171	123/170
Comparison of single detection for different lesions (positive/negative)	CNI group (n=63)	43/20	47/15	45/18
	CNII group (n=59)	34/25	32/27	35/24
	CNIII group (n=49)	27/22	24/25	26/23
	Total	104/67	103/68	106/65

**Table III:** Results of TCT combined with serum CA153 and CA50 on cervical cancer and precancerous lesions.

Group	Positive (n)	Negative (n)
Cervical cancer group (n=16)	15	1
Cervical precancerous lesion group (n=171)	135	36
Healthy group (n=106)	5	101
Total	155	138

**Table IV:** Comparison of efficacy by TCT, serum CA153 and CA50 alone or in combination.

Diagnostic methods	Sensitivity	Specificity	Accuracy	Positive predictive value	Negative predictive value
TCT	95.93%	59.41%	74.74%	62.03%	96.22%
Serum CA153	97.54%	60.23%	75.77%	63.64%	97.17%
Serum CA50	96.00%	60.12%	75.43%	63.10%	95.28%
TCT+ serum CA153+ serum CA50	96.77%	73.19%	85.67%	80.21%	95.28%

**Fig.1:**
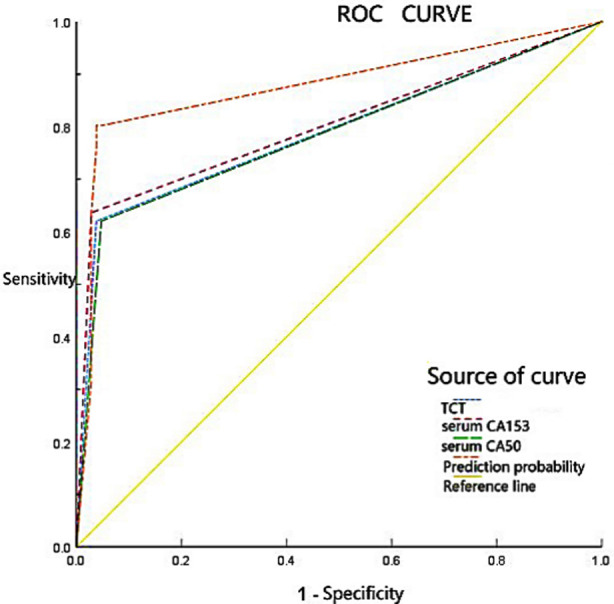
Diagnostic value of TCT, serum CA153 and CA50 in the detection of cervical cancer and precancerous lesions.

ROC curve showed that the area under the curve (AUC) of TCT and serum CA153 and CA50 in the detection of cervical cancer and precancerous lesions was 0.791, 0.864 and 0.787, respectively, and the AUC of combined detection of TCT and serum CA153 and CA50 in patients with cervical cancer and precancerous lesions was 0.877. Fig.1. The area under the ROC curve of the combined detection was significantly higher than that of the single detection (*p*< 0.05). Pairwise comparison of TCT detection with serum CA153 and CA50 detection showed no significant difference in the area under the ROC curve (*p*> 0.05). Table-V.

**Table V:** Comparison of the area under the ROC curve among the four detection methods.

Diagnostic methods	*Z*	*p*
Combined detection vs TCT	2.525	0.011
Combined detection vs serum CA153	2.192	0.028
Combined detection vs serum CA50	2.642	0.008
Serum CA153 vs TCT	0.360	0.719
Serum CA153 vs serum CA50	0.471	0.637
TCT vs serum CA50	0.109	0.913

## DISCUSSION

Cervical cancer, as one of the common malignant tumors of the reproductive system in gynecology, ranks fourth in the world in morbidity and mortality among gynecological malignancies. Early diagnosis and treatment are beneficial to effectively improve the survival rate of patients with cervical cancer.^11^-^13^ HPV infection is the key cause of cervical cancer. In the wake of the promotion and application of HPV vaccine in recent years, the early screening, diagnosis and treatment of cervical cancer have been ameliorated to a large extent.^14^,^15^ Given that tumor cells are complex and diverse and often have one or more markers, combined detection can improve the accuracy of early tumor screening.^16^,^17^ In this study, the value of TCT, serum CA153 and CA50 detection alone and combined detection for early diagnosis of cervical cancer and precancerous lesions is analyzed. It has been proved that the accuracy of combined detection is higher than that of single detection, which is of great significance for early clinical diagnosis of cervical cancer.

TCT diagnosis is carried out using cytology, which enables accurate staging of cervical cells, and most females are screened for cervical cancer with this detection method. Nevertheless, TCT diagnosis cannot detect gene changes in tumor cells, and misdiagnosis may occur only by cell morphology judgment, so TCT diagnosis alone will produce low sensitivity and specificity.^18^,^19^ In view of this, a combination of multiple methods is commonly used clinically to diagnose cervical cancer and its precancerous lesions. In this study, the effect of TCT on the diagnosis of cervical cancer and precancerous lesions was analyzed. By taking the pathological examination results as the gold standard, the sensitivity, specificity, accuracy, positive predictive value and negative predictive value of TCT alone in the detection of the three groups were 95.93%, 59.41%, 74.74%, 62.03% and 96.22%, respectively.

ROC curve showed that the AUC of TCT detection in patients with cervical cancer and precancerous lesions was 0.791, while TCT detection combined with serum CA153 and CA50 detection could significantly improve the accuracy, specificity and positive predictive value of early diagnosis of cervical cancer and precancerous lesions, suggesting that TCT detection could be used as an early diagnostic detection method of cervical cancer and precancerous lesions. However, TCT detection combined with other methods can achieve better results, which is of great significance for the early diagnosis of cervical cancer and precancerous lesions.

CA153 and CA50 are broad-spectrum tumor markers and have high clinical value in the diagnosis of a variety of tumors, among which CA153 is a commonly used tumor marker in breast cancer while CA50 is a tumor marker for digestive tract tumors.^20^,^21^ The levels of CA153 and CA50 are abnormally elevated not only in the serum of patients with breast cancer and colon cancer, but also in those with acute infectious diseases.^22^,^23^ Consequently, false positives or false negatives easily occur if CA153 or CA50 is used separately, thus affecting the early diagnosis of tumors. In contrast, when multiple detection methods are combined for screening diagnosis, the accuracy and specificity of diagnosis can be improved to a certain extent, and the incidence of misdiagnosis can be reduced.

In this study, the levels of serum CA153 and CA50 in the three groups were compared, and the levels of serum CA153 and CA50 in the cervical cancer group and the cervical precancerous lesion group were significantly higher than those in the healthy group (*p*<0.05). By taking the pathological examination results as the gold standard, the sensitivity, specificity, accuracy, positive predictive value and negative predictive value of serum CA153 detection alone in the three groups were 97.54%, 60.23%, 75.77%, 63.64% and 97.17%, respectively. The sensitivity, specificity, accuracy, positive predictive value and negative predictive value of serum CA50 detection alone in the three groups were 96.00%, 60.12%, 75.43%, 63.10% and 95.28%, respectively. The sensitivity, specificity, accuracy, positive predictive value and negative predictive value of TCT combined with serum CA153 and CA50 in the detection of cervical cancer and precancerous lesions were 96.77%, 73.19%, 85.67%, 80.21% and 95.28%, respectively. It can be seen that the combination detection boasts the efficacy of improving the specificity, accuracy and positive predictive value of the diagnosis of cervical cancer and precancerous lesions.

ROC curve showed that the AUC of serum CA153 and CA50 detection for cervical cancer and precancerous lesions was 0.864 and 0.787, respectively, and the AUC of patients with cervical cancer and precancerous lesions was 0.877 when combined with TCT, and the area under the ROC curve of combined detection was significantly higher than that of single detection. No significant difference was observed in the area under the ROC curve in the comparison between serum CA153 and CA50 detection alone. Serum CA153 and CA50 can be used as markers for the diagnosis of cervical cancer and precancerous lesions, and can significantly improve the diagnostic accuracy and positive diagnosis rate when combined with TCT, which is of great significance for the diagnosis of cervical cancer and precancerous lesions.

### Limitations of the study

Nevertheless, a small number of samples are included in this study, which may cause some deviations to the results. In response to this, proactive measures will be taken in the follow-up to increase the sample size for in-depth investigation.

## CONCLUSION

TCT combined with serum CA153 and CA50 is an effective method for the detection of cervical cancer boasting high accuracy. With TCT combined with serum CA153 and CA50 detection, a variety of effects can be achieved, such as effectively improving the value of early diagnosis of cervical cancer and precancerous lesions, significantly improving the diagnostic sensitivity, which is conducive to the clinical differentiation of cervical cancer and precancerous lesions.

### Authors’ Contributions:

**HL &** **SD:** Designed this study ,prepared this manuscript, are responsible and accountable for the accuracy and integrity of the work.

**LL &** **JS:** Collected and analyzed clinical data.

**HL:** Significantly revised this manuscript.
